# Relationship of cyberchondria to hypochondriac beliefs and internet use

**DOI:** 10.1192/j.eurpsy.2021.493

**Published:** 2021-08-13

**Authors:** S. Kumchenko, N. Rostovzeva, E. Rasskazova, A. Tkhostov

**Affiliations:** 1 Clinical Psychology, Moscow State University, Moscow, Russian Federation; 2 Faculty Of Psychology, Lomonosov MSU, Moscow, Russian Federation

**Keywords:** Internet, cyberchondria, hypochondriac beliefs

## Abstract

**Introduction:**

Although cyberchondria was suggested as a separate phenomenon (Starcevic, Berle, 2013, Starcevic, 2017), it is by definition related to both health anxiety, general hypochondriac beliefs and behavior and Internet use (Baumgartner and Hartmann, 2011, Eastin and Guinsler, 2006, Singh and Brown 2014).

**Objectives:**

The aim was to reveal relationship between cyberchondria in adult Internet users, Internet use and hypochondriac beliefs and behavior.

**Methods:**

126 adults (18-70 years old) filled The Cyberchondria Severity Scale (CSS, McElroy, Shevlin, 2014), checklist of activities about health online, Scale for Assessing Illness Behavior (Rief et al., 2001), Cognitions About Body and Health Questionnaire (Rief et al., 1998).

**Results:**

Compulsion, Distress, Excessiveness, Reassuarance Seeking scales are related to various health-related activities online including both specialized (medical web-sites) and non-specialized (Wikipedia) ones (r=.25-.48, p<.01). Compulsion is closely related to surfing in social networks (r=.41, p<.01), excessiveness – to viewing of illnesses-related pictures (r=.48, p<.01) and reassurance seeking – to reading of online reports (r=.47, p<.01). Cyberchondria is related both to health anxiety (r=.37), hypochondriac behavior (r=.19-.41), beliefs about autonomic sensations, bodily weakness, intolerance to sensations and somatosensory ampliphication (r=.25-.31).

**Conclusions:**

In general population, different aspects of cyberchondria seem to reflect health anxiety and hypochondriac beliefs but are differently related to different forms of online behavior including use of more or less specialized web-sites. Research is supported by the Russian Foundation for Basic Research, project No. 20-013-00799.

